# Microorganism and filamentous fungi drive evolution of plant synapses

**DOI:** 10.3389/fcimb.2013.00044

**Published:** 2013-08-15

**Authors:** František Baluška, Stefano Mancuso

**Affiliations:** ^1^IZMB, Department of Plant Cell Biology, University of BonnBonn, Germany; ^2^LINV, Department of Plant, Soil and Environmental Science, University of FlorenceSesto Fiorentino, Italy

**Keywords:** plant evolution, symbiosis, actin cytoskeleton, endocytosis, vesicle recycling, synapses, plant behavior, auxin transport

## Abstract

In the course of plant evolution, there is an obvious trend toward an increased complexity of plant bodies, as well as an increased sophistication of plant behavior and communication. Phenotypic plasticity of plants is based on the polar auxin transport machinery that is directly linked with plant sensory systems impinging on plant behavior and adaptive responses. Similar to the emergence and evolution of eukaryotic cells, evolution of land plants was also shaped and driven by infective and symbiotic microorganisms. These microorganisms are the driving force behind the evolution of plant synapses and other neuronal aspects of higher plants; this is especially pronounced in the root apices. Plant synapses allow synaptic cell–cell communication and coordination in plants, as well as sensory-motor integration in root apices searching for water and mineral nutrition. These neuronal aspects of higher plants are closely linked with their unique ability to adapt to environmental changes.

## Evolution of eukaryotic cells: life is inherently invasive, infective, and collaborative

After years of controversy, the endosymbiotic theory won the race and it is now widely accepted that eukaryotic cells emerged on the evolutionary scene after several endosymbiotic event(s) (Archibald, 2011). Although the nature of the host cells and the evolutionary origin of the nucleus are still hotly discussed, it is obvious that microorganisms shaped the evolution of eukaryotic cells (Margulis, 1981, 2001, 2004; Baluška et al., 2004a,b; Archibald, 2011; Vesteg and Krajcovic, 2011; Katz, 2012). Besides mitochondria, plant cells also have symbiotic plastids—this means presence of three independent but highly integrated genomes in one cell (Herrmann et al., 2003). Recent analyses of the available sequence data confirmed the earlier suspicion that Chlamydia bacteria had assisted in this further increase in the complexity of eukaryotic cell (Becker et al., 2008; Price et al., 2012a; Spiegel, 2012; Ball et al., 2013; Baum, 2013). Although both mitochondria and plastids lost their independence during this very long intracellular symbiosis, they still retained some microbial autonomy allowing them even to change their host cells (Spees et al., 2006; Acquistapace et al., 2011; Rebbeck et al., 2011; Islam et al., 2012; Prockop, 2012; Thyssen et al., 2012). Moreover, some microorganism-derived organelles, such as mitosomes and hydrogenosomes, lack a genome and any DNA (Dolezal et al., 2005; Shiflett and Johnson, 2010), suggesting that some other organelles (e.g., peroxisomes) might also have a microbial origin (De Duve, 2007; Duhita et al., 2010). The logic of biological evolution is related to the inherently invasive, infective, and collaborative nature of viruses, microorganisms, and other organismic units of prokaryotic life (Margulis, 1997, 2001, 2004; Baluška, 2009). Unfortunately, most of these ancient mergers and endosymbiotic events are fully obliterated by an inherent tendency of endosymbionts to lose their DNA, phenomenon related to the principle of biological attraction (Agnati et al., 2009), and to be transformed into membraneous compartments and organelles.

## Plant evolution: land invasion via bacterial and fungal alliances

**Figure 1:**
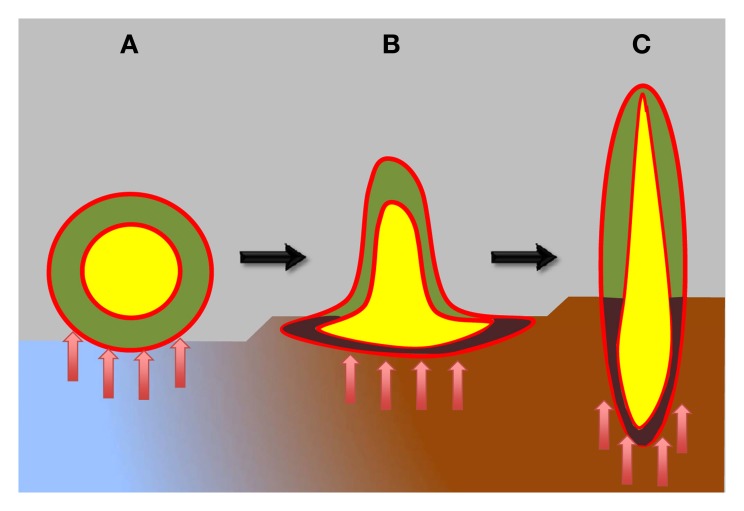
**Schematic view of plant body organization in plant evolution.** Hypothetical ancient plants are depicted as having atelomic body **(A)**, resembling lichen-like thallus, while still lacking true shoots and roots. These hypothetical ancient plants already possessed two types of cells: heterotrophic central cells (yellow), protected from the photosynthetic cells by ancient endodermis-like epithel tissue (red). The surface of these ancient plants was likewise protected by ancient epidermis-like epithel tissue (red). During evolution, ancient plants progressively developed shoots and roots **(B,C)**. Moreover, vascular systems with xylem and phloem parts, as well as the true epidermis and endodermis evolved (red). Epithelium-like lining, known as xylem and phloem parenchyma, covers xylem and phloem elements (not shown in this diagram) that integrate the whole plant bodies into well-coordinated unities. Blue-brown colors depict sea-land transition. Red arrows depict microbial interactions.

Ever since ancient plants invaded land, they have dramatically evolved from simple bodies lacking any sensory specification and organismal behavior to higher plants dominating the recent macro-flora. During higher plant evolution, plants drastically increased the complexity of their bodies, with recent angiosperms representing the most evolved plants. There were several waves of innovations concerning the organization of their bodies. The most ancient land plants are considered to be telomic, lacking root and shoot organization. The available fossil record indicates that roots evolved later than shoots and leaves, but the lower capacity of roots to fossilize may have resulted in a distorted fossil-based phylogenetic representation (Kenrick and Crane, 1997; Boyce, 2009). Therefore, it seems that the first roots, shoots, and leaves evolved together with the evolution of the first xylem and phloem elements, representing the vascular system. Roots and shoots, as well as vascular elements, followed an independent evolutionary path in vascular plants. The highest complexity of these organs was reached in angiosperms (flowering plants), that emerged much later in land plant evolution (Kenrick and Crane, 1997; Langdale, 2008; Boyce, 2009; Dolan, 2009). Generally, the evolutionary history of land plants is rich in examples of convergent evolution. The nature of this phenomenon is still not clear, though it might be related to high phenotypic plasticity, lateral gene transfer, and an abundant record of symbiosis (Agnati et al., 2009; Baluška, 2009, 2011). It is assumed that land plants evolved from algae (McCourt et al., 2004; Wodniok et al., 2011; Zhong et al., 2013), but the initial invasion of dry and rocky land was probably possible only through an alliance of ancient algae and fungi (Jorgensen, 1993; Selosse and Le Tacon, 1998; Bidartondo et al., 2011), forming lichen-like composite super-organisms. In fact, there are obvious similarities between the thallus forms of contemporary lichen, which can survive even on dry rocks, and of ancient telomic plant bodies (Heckam et al., 2001; Sanders, 2006; Figure 1). Only alliances of fungi, algae, and bacteria could allow for the shift from ocean to hostile land environments, where progressive transformation led to emergence of fertile life-supporting land. Microorganisms and filamentous invasive fungi were essential for the chemical weathering of minerals, which was, in turn, a crucial prerequisite for the appearance and evolutionary transition of the first ancient land plants into highly specialized modern higher plants (Jorgensen, 1993; Kenrick and Crane, 1997; Selosse and Le Tacon, 1998; Langdale, 2008; Boyce, 2009; Dolan, 2009; Bidartondo et al., 2011). These alliances between plants, filamentous fungi, algae, and microorganisms are also obvious in the current plants (Bonfante, 2003; van der Heijden et al., 2008; Baluška, 2009; Bonfante and Anca, 2009; Jansa et al., 2013).

## Feedback cycles between root exudates and microorganisms shape rhizosphere

The root-soil interface, also known as the rhizosphere, is a complex habitat, which is essential for the plant's well-being and survival in challenging underground environments (Watt et al., 2006; Badri et al., 2009; Berendsen et al., 2012; Kumar and Bais, 2012; Mendes et al., 2013). This unique ecosphere represents one of the most energy-rich habitats on Earth (McCully, 1999; Watt et al., 2006; Berg and Smalla, 2009; Bisseling et al., 2009; Bakker et al., 2013). Plants invest about 20% of their photosynthetically fixed carbon into feeding the rhizosphere microbiome and other organisms living in this unique ecosphere (Odell et al., 2008; Bisseling et al., 2009). Root tips are most active with respect to feeding the rhizosphere, with the largest carbon depositions situated around the first 3 mm of maize roots (Odell et al., 2008), which include—besides the root cap—the meristem and the transition zone (Baluška et al., 2010b). The release of infochemicals relevant to organismal communication promotes both interkingdom communication (Badri et al., 2009; Witzany, 2012) and plant-plant communication (Walker et al., 2003; Bais et al., 2004).

## Auxin as interkingdom signaling molecule allowing communication between plants, filamentous fungi and microorganisms

The plant hormone auxin is an important player for interkingdom communication in the rhizosphere. It is not only a crucial signaling molecule for plant biology, but it is also an ancient signaling molecule used by microorganisms (Lambrecht et al., 2000; Pii et al., 2007; Spaepen et al., 2007; Mazhar et al., 2013). Auxin acts as a bacterial (Spaepen and Vanderleyden, 2011) and a fungal (Gruen, 1959; Ulrich, 1960; Splivallo et al., 2009; Tanaka, 2009) signaling molecule, facilitating the evolution of interkingdom communication (Badri et al., 2009; Ortiz-Castro et al., 2011; Witzany, 2012). As a consequence of the polar auxin transport in plants, auxins derived from bacteria and filamentous fungi living in the rhizosphere initiate several growth and developmental processes such as root hair initiation and tip growth, lateral root formation, and the plasticity of root system architecture (Contreras-Cornejo et al., 2009; Splivallo et al., 2009; Zamioudis et al., 2013).

## Auxin and neurotransmitters control root system architecture

In 2003, we proposed that polar auxin transport at the root apex is accomplished through a neurotransmitter-like mode, with auxin being secreted via an endocytic vesicle recycling process across the plant-specific synapses of root apices (Baluška et al., 2003). This scenario has been further supported by findings that the polar auxin transport in Arabidopsis root apices is mainly based on active vesicle recycling of PINs rather than the mere presence of PINs at the plasma membrane polar domains (Li and Xue, 2007; Mancuso et al., 2007; Shen et al., 2008; Yang et al., 2008). Moreover, serotonin, tryptophan-derived transmitter conserved in plants and animals, is structurally similar to auxin (Pelagio-Flores et al., 2011). In addition to serotonin, L-glutamate, and acetylcholine are also known to regulate root growth and root system architecture, the latter as a ligand of plant glutamate receptor-like channels (Sagane et al., 2005; Walch-Liu et al., 2006; Sugiyama and Tezuka, 2011; Price et al., 2012b; Forde et al., 2013; Vincill et al., 2013). During plant sexual reproduction, communication between the male gametophyte and the female pistil tissue has been shown to be mediated by the amino acid D-serine via GLRs; this is strongly reminiscent of neuronal synaptic communication in animals (Michard et al., 2011). Importantly, both L-glutamate and serotonin are root-specific in their control of development and phenotypic plasticity of plants (Walch-Liu et al., 2006; Pelagio-Flores et al., 2011; Forde et al., 2013; Vincill et al., 2013). Interestingly, GLR3.3 localizes to the synaptic cross-walls of the Arabidopsis root apex transition zone (Vincill et al., 2013). Our preliminary data suggest that L-glutamate and GLRs control endocytic vesicle recycling (synaptic activity) in these root apex cells (Weiland Matthias et al., unpublished data). GLR3.3 is further relevant to root gravitropism (Miller et al., 2010) and controls calcium transients during action potentials induced by L-glutamate (Qi et al., 2006; Felle and Zimmermann, 2007; Li et al., 2013).

## Evolution of plant synapses: from ABP1 to synaptic PINs

**Figure 2:**
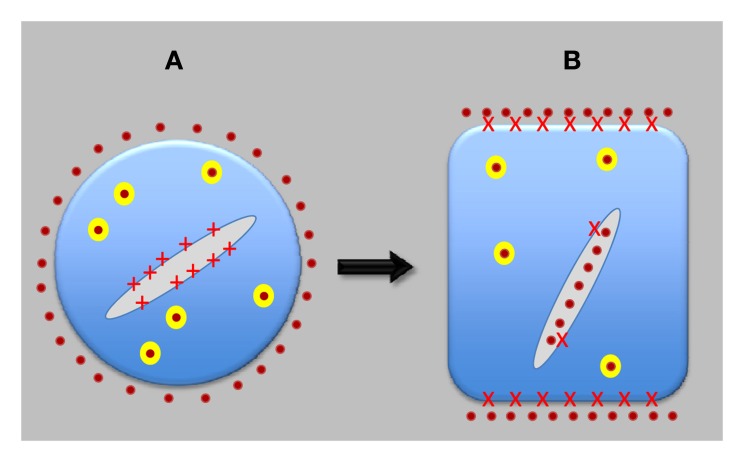
**Evolution of neuron-like plant cells.** Auxin-transporting synapses evolved only after plant cells transferred most of PINs from the endoplasmic reticulum (ER) to the plasma membrane (PM) and, in the opposite direction, most of ABP1 from extracellular space to the ER lumen. ABP1 then got access to cell periphery only in a strictly controlled manner and started to act as a central organizer of auxin-transporting synapses. Both PINs and ABP1 are integrated with sensory systems and also control motor responses of root apices. **(A)** Shows pre-synaptic cell, and **(B)** shows synaptic cell, as found in the root apices of contemporary higher plants. Red crosses depict APB1; yellow balls are recycling vesicle; and orange dots represent auxin.

In vascular plants, auxin-binding protein 1 (ABP1) binds auxin at the outer face of the plasma membrane. However, most ABP1 is confined within the endoplasmic reticulum (ER), where the KDEL sequence retrieves ABP1 from the cis Golgi back to the ER (Napier et al., 1992). The fact that *Physcomitrella patens* ABP1 lacks this KDEL-based ABP1 retrieval mechanism (Panigrahi et al., 2009) implies that ancient ABP1 was not enriched within ER (Figure 2). This conclusion is relevant to our understanding of plant synapse evolution. Plant synapses evolved together with the vascular system and the polar auxin transport machinery based on plant-specific PINs (Friml, 2003; Paponov et al., 2005; Tromas et al., 2010). PINs participate in the highly polar cell-to-cell transport of auxin, which is essential for plant development (Friml, 2003), sensory perception, as well as for sensory-motor circuitry underlying plant tropisms (Chen and Masson, 2005; Paponov et al., 2005; Mancuso et al., 2007; Baluška et al., 2010b; Langowski et al., 2010; Tromas et al., 2010).

Auxin binding to ABP1 at the outer leaflet of the plasma membrane induces hyperpolarization and action potentials (Barbier-Brygoo et al., 1989; Felle et al., 1991). These ABP1-mediated electrical responses to auxin also induce physico-chemical changes in the plasma membrane, as evidenced by the loss of fluorescence of the endocytic tracer synapto-Red reagent (FM4-64) (Dahlke et al., 2010). Interestingly in this respect, FM4-64, known as synaptored, accumulates at both brain and plant synapses (Baluška et al., 2003, 2005, 2010b; Mancuso et al., 2007; Baluška, 2010).

Recently, ABP1 has been shown to support high rates of clathrin-mediated endocytosis at plant synapses in roots (Robert et al., 2010), which is linked to the permanent character of the trans-Golgi network (TGN) in transition zone cells (Šamaj et al., 2005; Kang, 2011; Wang et al., 2013). This feature of root cells active in synaptic vesicle recycling is similar to neuronal cells having active synapses enriched with the so-called Golgi Outposts (Baluška, 2010; Baluška et al., 2010b; Schecterson et al., 2010; Lewis and Polleux, 2012; Ori-McKenney et al., 2012). It is noteworthy that those root apex cells which have low activity of endocytosis and high activity of exocytosis, such as secretory root cap cells or elongating root cells, lose their TGNs as independent organelles via their active secretion (Baluška, 2010; Baluška et al., 2010b). This is the reason why secretory root cap cells as well as rapidly elongating cells do not generate large BFA-induced compartments (Kang, 2011). In the *abp1* mutant lines, there is a general inhibition of endocytosis and even transition zone cells exhaust their TGNs (Robert et al., 2010). Therefore, BFA does not cause formation of large BFA-induced compartments in root apex cells of *abp1* mutant line. Besides underlying high rates of endocytosis, synaptic ABP1 transmits signals from the plant-specific neurotransmitter auxin, released by the adjacent synaptic cell partner and traversing the synaptic cleft. The binding of auxin to ABP1 on the plasma membrane of adjacent cells has three fundamental effects—it: (1) induces electric responses (Barbier-Brygoo et al., 1989; Felle et al., 1991; Dahlke et al., 2010), (2) inhibits the ABP1-mediated clathrin based endocytosis (Robert et al., 2010), and (3) induces very rapid (within 30 s) activation of plant Rho GTPases Rop2 (Xu et al., 2010).

During evolution, plasma membrane PIN transporters evolved from the ER located PINs in land plants (Mravec et al., 2009; Xu et al., 2010), together with plasma-membrane-associated ABP1. The auxin receptor ABP1 is recovered from secretory pathways by the KDEL peptide which bring it back to the ER (Napier et al., 2002; Tromas et al., 2010). This afforded ABP1 only a limited and highly regulated access to the plasma membrane (Tromas et al., 2010) and the KDEL system, therefore, highly selectively regulates the transport and distribution of plasma-membrane-associated ABP1. The small concentrations of ABP1 incorporated into the plasma membrane integrate auxin transport with clathrin-based endocytosis (Tromas et al., 2010). This process helps to control the synaptic activity driven by endocytic vesicle recycling between the polar synaptic domains of the plasma membrane and TGN/early endosomes (Baluška et al., 2002, 2010b; Šamaj et al., 2005; Baluška, 2010; Xu et al., 2010; Zárský and Potocký, 2010; Kang, 2011; Viotti et al., 2011).

## Evolution of plant synapses: expansion of synaptic PINs during plant evolution

As mentioned, key evolutionary innovations of vascular plants—the formation of vascular system and true roots—were associated with the invention of plasma membrane-associated PINs that exported auxin out of cells (Krecek et al., 2009; Mravec et al., 2009; Tromas et al., 2010). This allowed synaptic communication through signal-mediated release of auxin into the synaptic space between two adjacent cells connected via a synaptic cell–cell adhesion domain (Figure 2). Besides increasing the number of synaptic PINs, which is higher in more evolved monocot species such as maize, rice, and Sorghum in comparison with dicot species such as Arabidopsis (Krecek et al., 2009; Wang et al., 2009; Shen et al., 2010), the highest number of synaptic PINs is active in root apices where two inverted fountains of polar auxin transport determine the formation and maintenance of the transition zone (Baluška et al., 2005, 2010b). The monocot-specific PINs of classes 9 and 10 are expressed in root apices too, and prove to be involved in the formation and development of adventitious roots (Wang et al., 2009; Shen et al., 2010). The complexity of root systems is higher in monocots than in dicots (Hochholdinger and Zimmermann, 2008), which indicates that plants and roots continue to evolve very rapidly.

## Plant epithels, epithelial synapses, and host-pathogen vs. symbiotic synapses

The evolution of roots is closely linked to that of plant vascular systems and of flowers. Roots, vascular systems, and flowers represent relatively late plant innovations and contribute substantially to the complexity of plant bodies after the colonization of land by terrestrial plants. Importantly, all three features are inherently associated with polar auxin transport, underlying their likely co-evolution. Invasive vascular systems spread throughout the plant body, but are most prominent in roots where they are organized into central cylinder (stele), which is enclosed in an epithelium-like endodermis (Alassimone et al., 2011). This so-called “inner skin” of roots shows many features resembling animal epithelia (Roppolo et al., 2011), suggesting the view of the endodermis as a plant-specific epithelium. Casparian strips of root endodermis resemble tightly arranged junctions of animal epithelia, while the proteins responsible for their formation, CASPs (*Ca*sparian *S*trip membrane domain *P*roteins), show similarities to CLAUDINs of the tight junctions of animal epithelia (Roppolo et al., 2011). In fact, epithelial tight junctions are enriched with synaptic proteins and act as epithelial synapses for cell–cell communication (Tang, 2006; Yamada and Nelson, 2007). Besides endodermis, epithelium-like characteristics are also obvious in the root epidermis (Langowski et al., 2010), as well as in the epithelium-like cell lining of xylem and phloem elements. Importantly, the invasive fungal-like vascular central cylinder (stele) reaches up to the very apex of roots, when phloem elements protrude up to the transition zone, while the endodermis reaches up the very root apex. This location of sucrose unloading phloem allowed evolution of the transition zone (brain-like command center). In contrast, the vascular central cylinder and sucrose unlading phloem elements are missing from the very shoot apices. It can be proposed that numerous invasions of ancient roots via bacteria and especially fungi resulted in generation of abundant host-pathogen synapses which transformed later into the symbiotic and, finally, into the auxin-secreting root synapses most active in epithel-like epidermis and endodermis at and around the transition zone. Emerging vascular systems, especially phloem, played central role in evolution of root apex transition zone specialized for synaptic activities and for the sensory-motor nature of the root apex.

## Evolution of auxin-secreting synapses in higher plants

**Figure 3:**
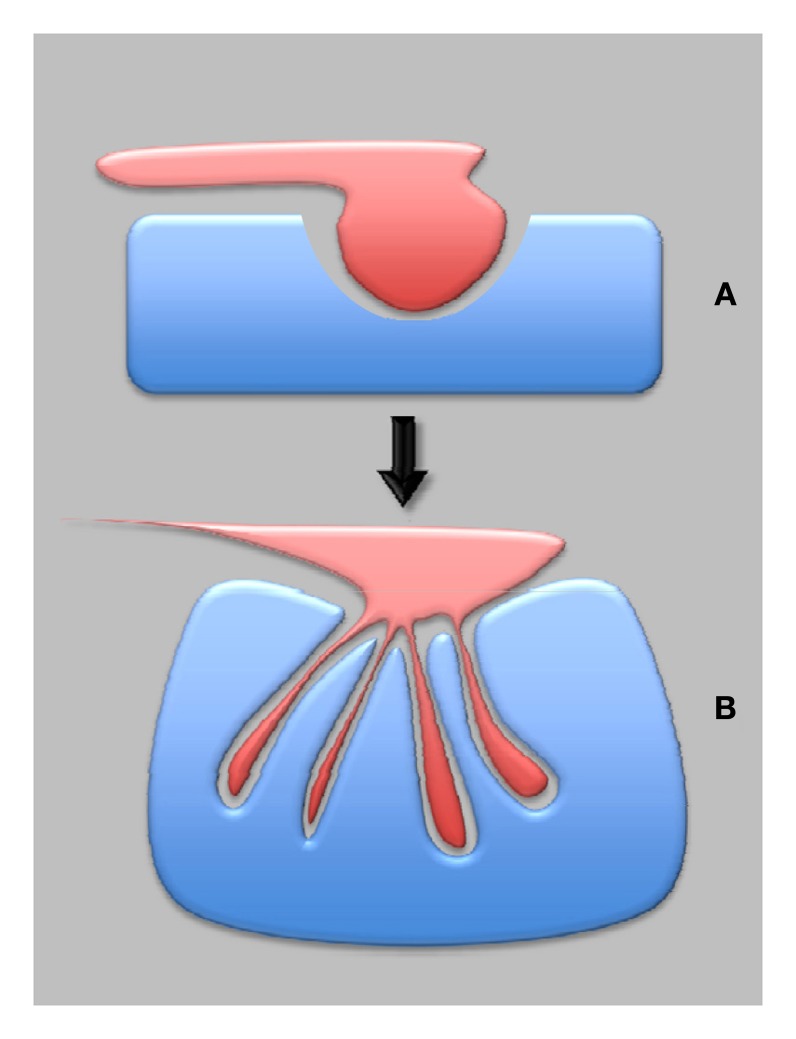
**Plant synapses in plant evolution—from pathogenic to symbiotic synapses.** The most ancient are proposed to be pathogenic synapses **(A)**, which eventually developed into the symbiotic synapses **(B)**, provided that both partners negotiated well-balanced interactions.

**Figure 4:**
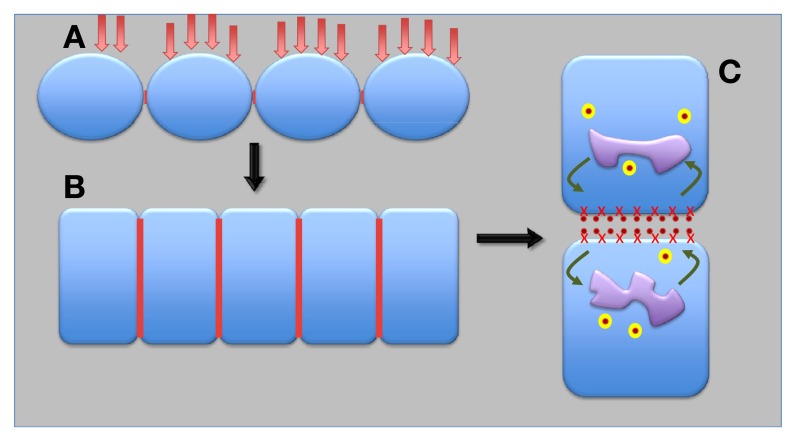
**Possible scenario for evolution of epithelial and auxin transporting plant synapses.** Under repeated pathogen attacks and with progressive exposures of ancient terrestrial plants to dry environments, ancient surface epithel-like tissue **(A)** developed into the contemporary epidermis and endodermis plant epithels **(B)**. Finally, auxin-transporting synapses evolved from epithelial synapses **(C)**. Red lines show synaptic cell–cell adhesion domains; red crosses depict APB1; yellow balls are recycling vesicles; and orange dots represent auxin.

Increased auxin, calcium, and inositol trisphosphate (IP3) levels in root cells shift the usual rootward PIN polarity to the shootward polarity (Zhang et al., 2011). Fungal invasions increase auxin levels in cells of root apices and drive lateral root primordia formation (Felten et al., 2009; Splivallo et al., 2009). PIN2 is unique among other PINs with its rootward polarity in the cortex cells of the meristem, even though it switches into the shootward polarity in the transition zone (Chen and Masson, 2005; Rahman et al., 2010), as is the case in all epidermis cells. One possible evolutionary scenario explaining why PIN2 has an opposite polarity is that repeated fungal invasions of roots resulted in increased auxin levels in epidermis and cortex cells, switching the rootward PIN polarity into the PIN2-specific shootward polarity. This would imply that symbiotic synapses, evolved from the more ancient pathogenic synapses (Baluška et al., 2005; Kwon et al., 2008; Lima et al., 2009; Rahman et al., 2010). Symbiotic synapses represent predecessors for neuronal-like auxin secreting synapses of the transition zone (Figure 3) that drive not only sensory-motor based root behavior (Figure 4) and phenotypic plasticity of plants, but perhaps also the cognitive and intelligent nature of higher plants (Trewavas, 2005, 2009; Baluška et al., 2009; Calvo Garzón and Keijzer, 2011; Karpiński and Szechyńska-Hebda, 2011; Trewavas and Baluška, 2011).

## Sensory basis of intelligence in higher plants

The body of flowering plants has a clear polarity, with the root apices forming the sensory pole, specialized in searching for water and mineral nutrients, and the shoot apices forming the reproductive pole, specialized in sexual reproduction (Baluška et al., 2006; Baluška and Mancuso, 2009a,b). The heterogeneous and patchy nature of soils, when nutritionally rich patches are located close to nutritionally poor and dry (or even toxic) soil portions, presents a difficult challenge for roots (Shemesh et al., 2010) in their major task of finding and acquiring enough nutrition so as to feed the whole plant. Plants overcome some of these challenges by improving their capacity to locate and obtain foods through sensory experience (Trewavas, 2005, 2009; Baluška et al., 2009; Barlow, 2010a,b). In addition, plants grow in dense populations, often resulting in fierce underground competition among roots from neighboring competing plants (Novoplansky, 2009). Roots discriminate *self* from *non-self* roots and they are also capable of kin recognition (Gruntman and Novoplansky, 2004; Biedrzycki et al., 2010) and swarm intelligence (Baluška et al., 2010a; Ciszak et al., 2012). Sensory discrimination and coordinated action enable plants, especially their roots, to secure territories for optimal plant survival and reproduction. While the cellular and molecular biology behind these processes is still poorly understood, the capacity of plants to repeatedly sense and adapt to environmental conditions in a manner that selectively optimizes their own individual ecological fitness and/or that of their cohorts demonstrates that plants exhibit primitive forms of intelligence still ascribed to animals (e.g., Romanes, 1884).

## Evolutionary origin of plant behavior and intelligence

In the evolutionary history of land plants, there is a clear tendency toward an increased sophistication of plant behavior (Trewavas, 2005, 2009; Brenner et al., 2006; Baluška and Mancuso, 2009a,b; Karban and Shiojiri, 2010). For instance, as noted above, complex plant bodies of modern plants are well integrated via long-distance signaling and communication (Baluška, 2013) to effect and coordinate behaviors important for survival, such as collective defenses against predators and foraging for soil nutrients, and reproduction, such as pollinator attraction or seed dispersal. To properly execute these sorts of sophisticated behaviors, plants require social (cooperation, competition, etc.) and cognitive-like abilities (learning, memory, perception, etc.) to limit and correct errors in signal detection and performance, among other phenomena (Trewavas, 2003). Such highly integrated social and cognitive-like traits presumably evolved in plants to allow them to interact with other plants, microorganisms, and even higher animals, including us humans (Trewavas, 2005, 2009; Brenner et al., 2006; Baluška and Mancuso, 2009a,b; Karban and Shiojiri, 2010; Calvo Garzón and Keijzer, 2011; Trewavas and Baluška, 2011). As previously discussed, significant aspects of plant structure and function which contribute to intelligent plant behavior can be traced to early evolutionary relationships with pathogenic and symbiotic microorganisms and fungi. These plant innovations, including synaptic machinery, are a putative direct outcome of intelligent-like reciprocation between plants and microorganisms to achieve selfish and selfless goals. Although we are now just starting to understand the communicative and intelligent nature of higher plants, similar social reciprocation appears endemic in plant-animal interactions as well. Many plants successfully manipulate animals for their purposes (Pacini et al., 2008; Baluška and Mancuso, 2009a,b). Higher plants must form very good internal models (e.g., networked somatic, genetic, and epigenetic computational systems capable of perceptual representation, memory and good predictions) concerning the biological nature of animals to accomplish any meaningful level of mutualism, parasitism, or commensalism, and co-evolution (Baluška and Mancuso, 2009a,b). Flowering plants seem to have convergently evolved these kinds of traits by co-inhabiting niches equally important to insects and higher animals. For example, animals primarily co-evolved with plants to serve as pollinators, but additional “services” also provide valuable ecological functions, such as seed dispersal, protection against parasites, and predators, as well as cultivation and propagation of crop plants around the globe by humans (Pollan, 2001). In order to attract relevant animals to perform services, flowering plants evolved a generous battery of strategies. The most efficient of these strategies is to supply animals with tasty, energy-rich foods in the form of nectars, seeds, and buds (Pollan, 2001). Moreover, plants evolved and continue to use sophisticated chemical communication, which allows them to exchange, both effectively and privately, survival-relevant information (Schiestl, 2005; Loivamäki et al., 2008; Baluška and Ninkovic, 2010; Dicke and Baldwin, 2010; Heil and Karban, 2010). Expert use of secure chemical signals, similar to chemical signaling by intelligent microorganisms (Crespi, 2001; Ben-Jacob et al., 2004; Bennett and Dunny, 2010), permits plants to perpetrate social altruism and cheating. Such phenomena can be found for the most evolved plants—the prominent monocot crop plants (maize, rice, wheat, barley) which entered co-evolution with humans—and orchids which evolved deceptive behaviors to attract pollinating animals to minimize ecological tradeoffs (Schiestl, 2005). Orchids, such as Ophrys, exemplify plant social deception in plant-animal interactions. They offer no nectar, which is energetically costly to synthesize, but rather attract and seduce male bees with perfect shapes, colors and scents, mimicking female bees ready for copulation (Schiestl, 2005). These smart orchids consequently gain significant bioenergetic advantages over competing flower plants, while exploiting animals for selfish benefits via sophisticated strategies.

## From microbial conscious cells to plant consciousness?

Consciousness is essential for life that entails intelligent responses to environmental challenges, with which all organisms are confronted. The idea of proto-consciousness, -intelligence, -cognition and -communication in microbes (Margulis, 2001; Ben-Jacob et al., 2004, 2006; Lowery et al., 2008; Ben-Jacob, 2009) remains controversial, although it is beginning to achieve broader acceptance within the scientific community. To what end these possible characteristics of microorganisms advanced plant intelligence is unknown. However, if intelligence and/or consciousness is expressed at the level of single cells, then multicellularity, whether it be associated with microorganisms, plants, or animals, can be reasonably expected to imbue higher degrees of consciousness in healthy organisms. It should be noted that the phenomenon of consciousness is a hypothetical construct with serious flaws contributing to our less than perfect understanding about its nature in humans (Clark, 2012; Clark and Hassert, 2013). In view of these conditions, we speculate that plant-specific consciousness allows higher plants to behave in an intelligent manner in order to optimize their coping with environmental challenges and diverse stress situations (Trewavas, 2003, 2005, 2009; Trewavas and Baluška, 2011). We offer as indirect and preliminary support of this notion the well-known findings that all organisms, including plants, are sensitive to anesthetics (Milne and Beamish, 1999; Eckenhoff, 2008; De Luccia, 2012). Moreover, intriguingly, stressed and wounded plants produce the powerful anesthetics ethylene and divinyl ether (Luckhardt and Carter, 1923; Powell et al., 1973; Campagna et al., 2003; Fammartino et al., 2007), perhaps as a means to attenuate plant-specific pain perceptions of stressed and wounded plants, allowing effective survival of sessile plants.

## Outlook

The evolution of land plants, especially the sudden appearance of flowering plants, represents one of the great mysteries for evolutionary theory. Indeed, Charles Darwin characterized this sudden origin and rapid evolution of flowering plants as the “abominable mystery” (Friedman, 2009). In addition, in his influential book published together with his son Francis, Charles Darwin proposed that the root apex acts as a brain of lower animals (Darwin, 1880; Baluška et al., 2009). The evolution of roots is closely interlinked with that of the vascular system and of the flowers, suggesting that plant body complexity was a relatively late acquisition, made after the land colonization by plants. Importantly, all three novel features are inherently associated with the polar auxin transport (Friml, 2003), and they obviously evolved simultaneously. We argue here, based on evidence from polar auxin transport, that early interactions with symbiotic and parasitic microorganisms helped force emergence and adaptation of plant vascular and root systems, particularly synaptic elements used for cell–cell communication, nutrient sensing and collection, and other behaviors. Subsequent terrestrial plant evolution has created and continues to create an increased vascular and root system complexities, that invests plants with purported plant-specific neuronal systems needed for the execution of adaptive goal-directed behaviors. Such goal-directed behaviors include cooperative and competitive social strategies that help plants obtain survival and reproductive advantages. While microorganisms may have played a protracted major role in driving plant evolution, the co-evolution of flowering plants and animals is not without impact.

Indeed, the most advanced in this respect are our crop plants, especially maize and rice, that entered into active co-evolution with humans some 10,000–15,000 years ago. It is essential to understand sensory, communicative, and cognitive complexity of the crop plants in order to cope with future challenges in human evolution. It seems that this will require a detailed analysis of auxin-secreting plant synapses, which underlie adaptive root and plant behavior.

### Conflict of interest statement

The authors declare that the research was conducted in the absence of any commercial or financial relationships that could be construed as a potential conflict of interest.
